# Supplementary material for: Pyrin inflammasome-driven erosive arthritis caused by unprenylated RHO GTPase signaling
Source: EMBO Mol Med. 2025 Aug 29;17(10):2691–712. doi: 10.1038/s44321-025-00298-0 (PMC12514176; doi:10.1038/s44321-025-00298-0)

Figure 1A ***Pggt1b***$^{+/+}$ ***Il1r*** $^{+/+}$

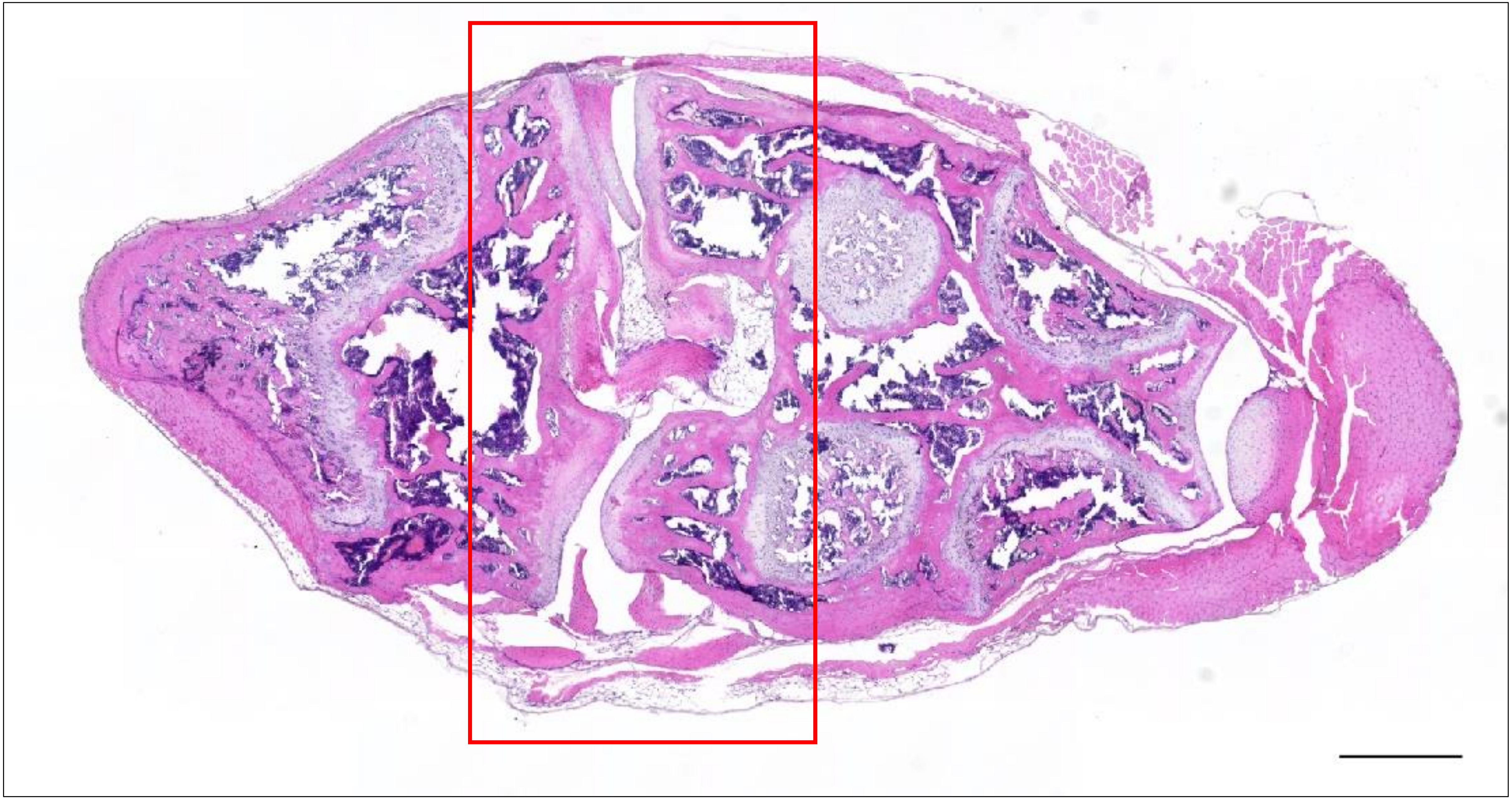

Figure 1A

Pggt1b$^{\Delta/\Delta}$ *Il1r* $^{+/+}$

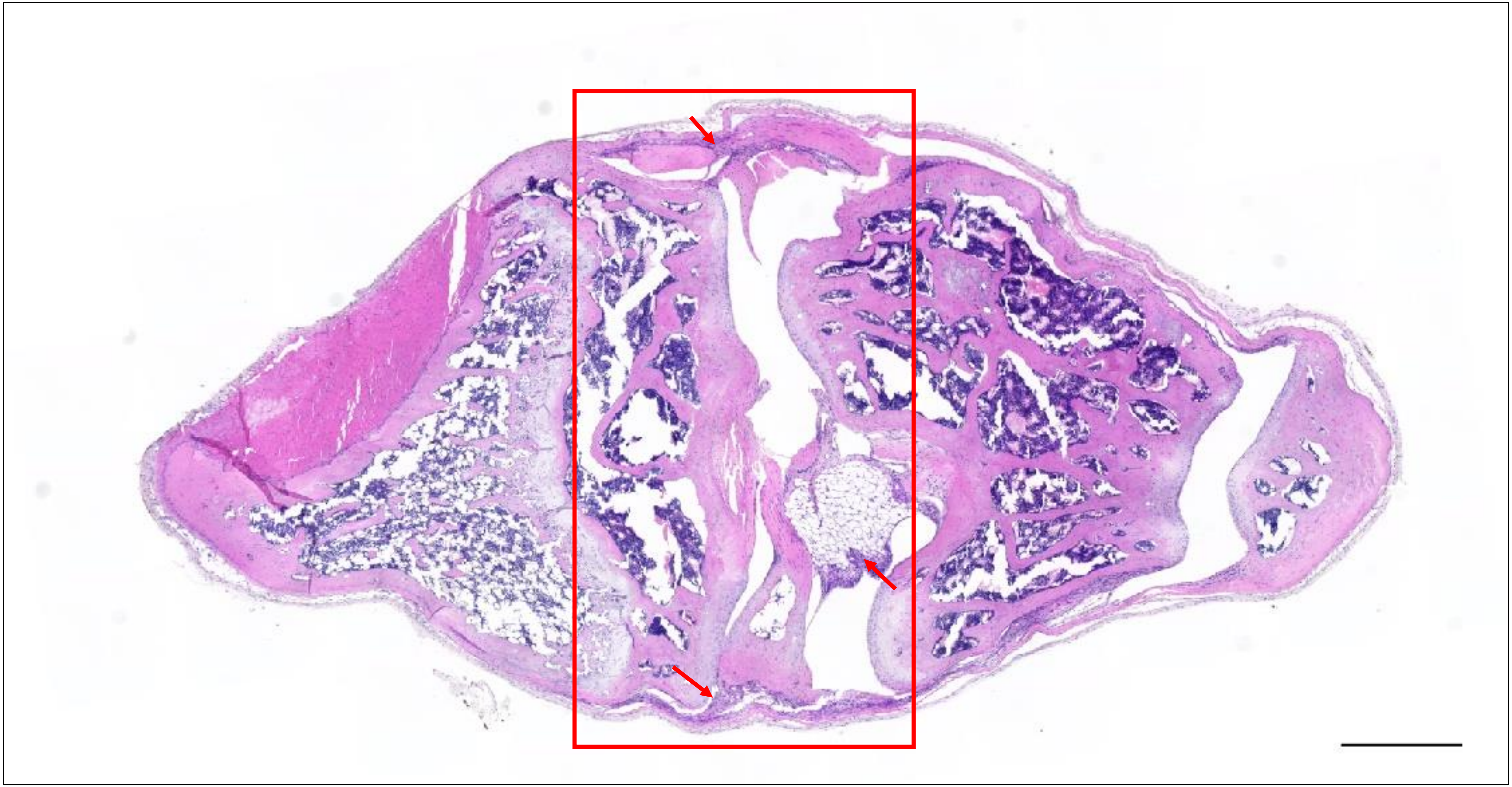

Figure 1A

Pggt1b$^{\Delta/\Delta}$ *Il1r* $^{-/-}$

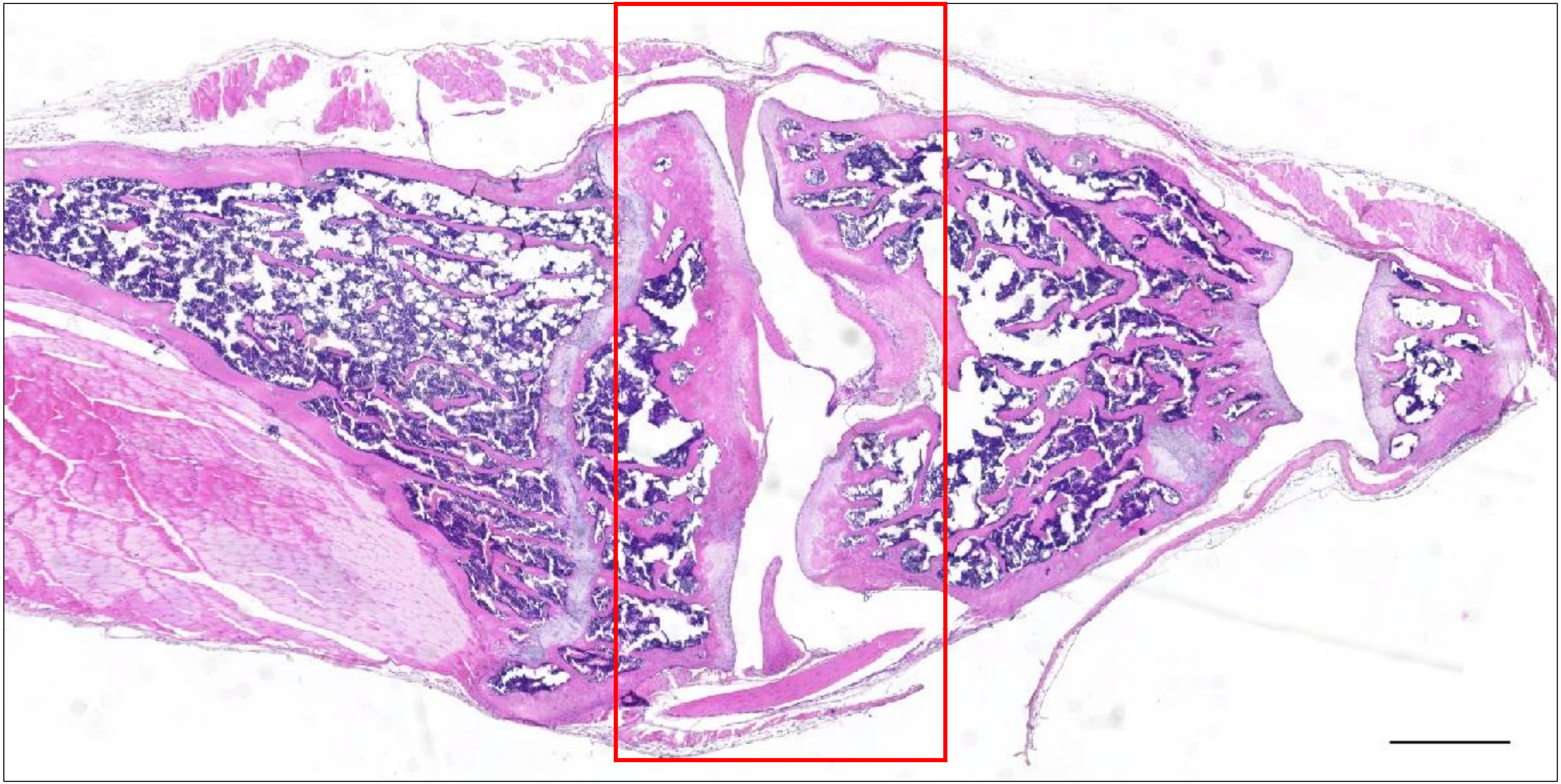

Supplement: Supplementary file 2 — Source data Fig. 1 [file 44321_2025_298_MOESM2_ESM.zip › Source Data_Figure 1/1A/SD1A.pdf]
