# Supplementary material for: Pyrin inflammasome-driven erosive arthritis caused by unprenylated RHO GTPase signaling
Source: EMBO Mol Med. 2025 Aug 29;17(10):2691–712. doi: 10.1038/s44321-025-00298-0 (PMC12514176; doi:10.1038/s44321-025-00298-0)

Figure 4A ***Pggt1b***$^{+/+}$ ***Asc*** $^{+/+}$

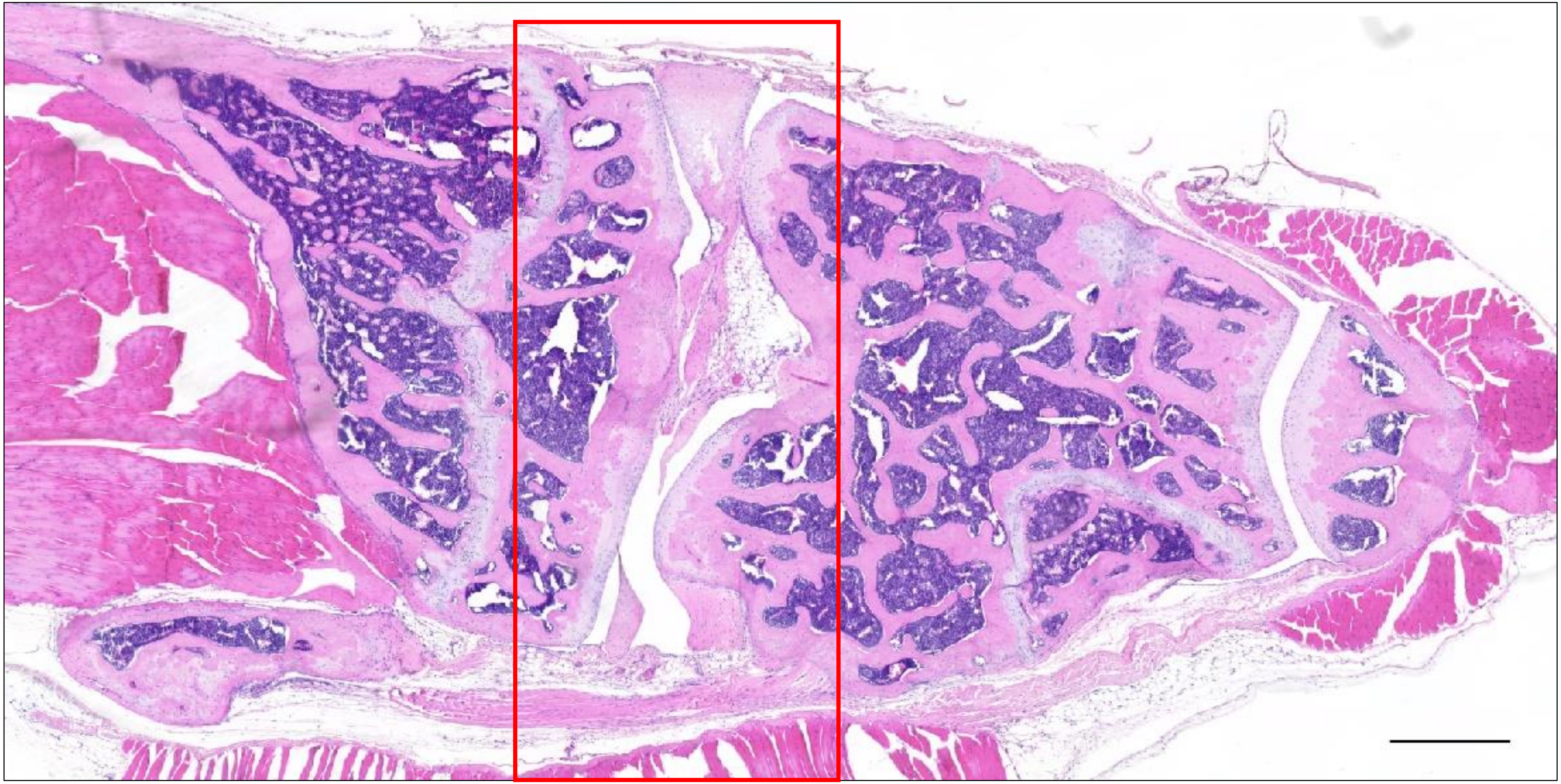

Figure 4A *Pggt1b*$^{\Delta/\Delta}$ *Asc*$^{+/+}$

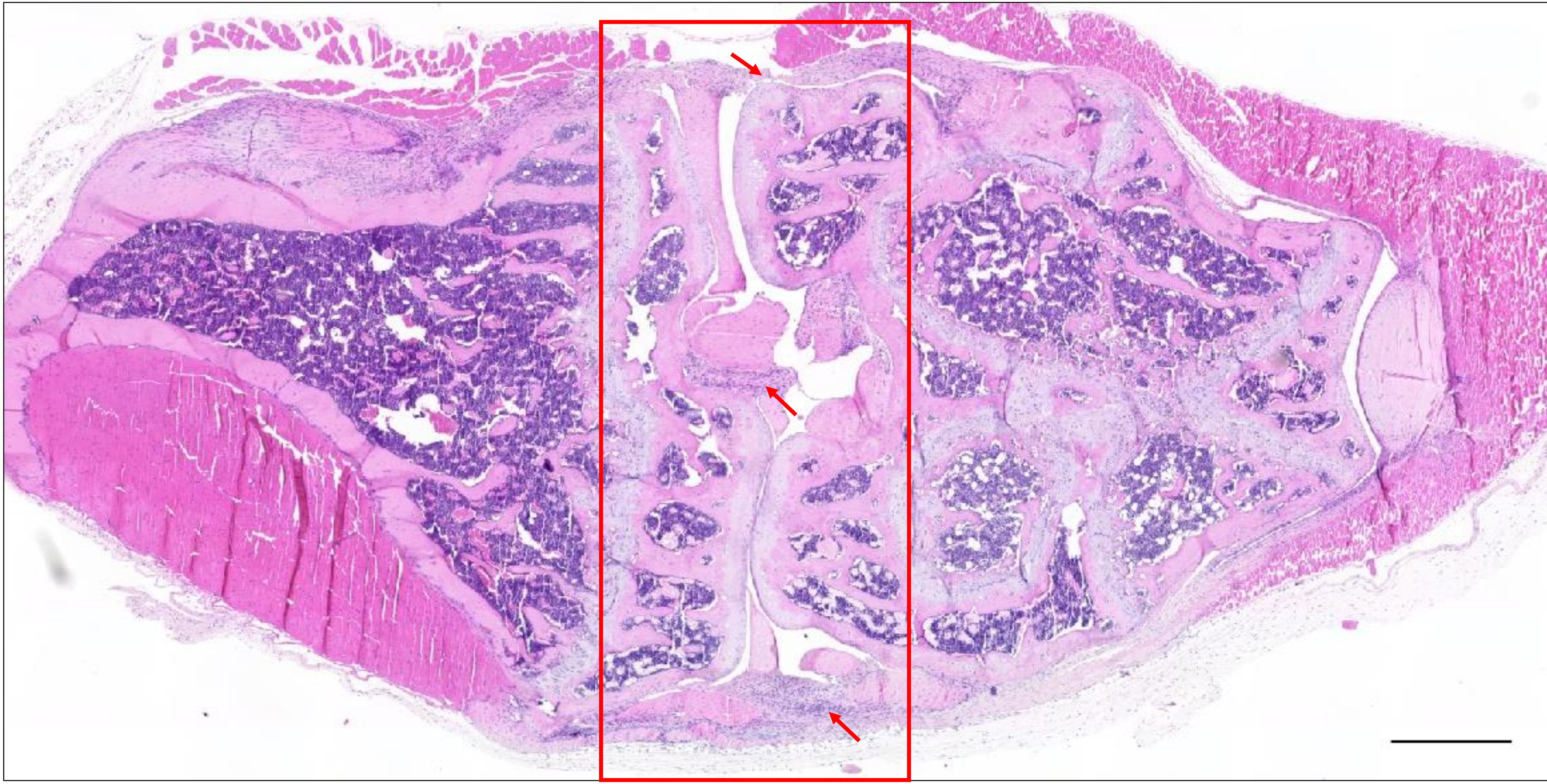

Figure 4A *Pggt1b*$^{\Delta/\Delta}$ *Asc*$^{-/-}$

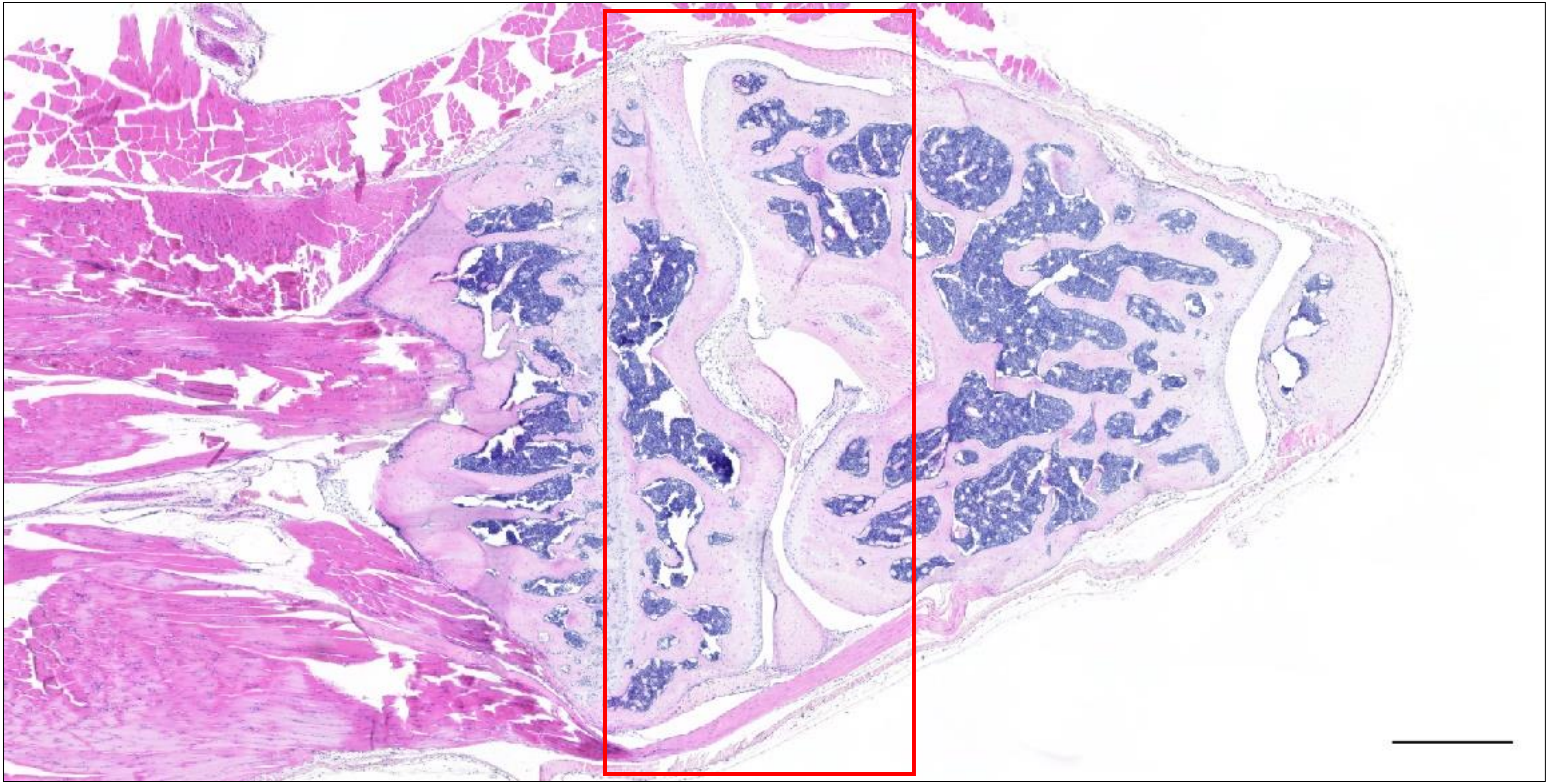

Supplement: Supplementary file 5 — Source data Fig. 4 [file 44321_2025_298_MOESM5_ESM.zip › Source Data_Figure 4/4A/SD4A.pdf]
