# Supplementary material for: Pyrin inflammasome-driven erosive arthritis caused by unprenylated RHO GTPase signaling
Source: EMBO Mol Med. 2025 Aug 29;17(10):2691–712. doi: 10.1038/s44321-025-00298-0 (PMC12514176; doi:10.1038/s44321-025-00298-0)

Figure 4G ***Pggt1b***$^{+/+}$ ***MefV*** $^{+/+}$

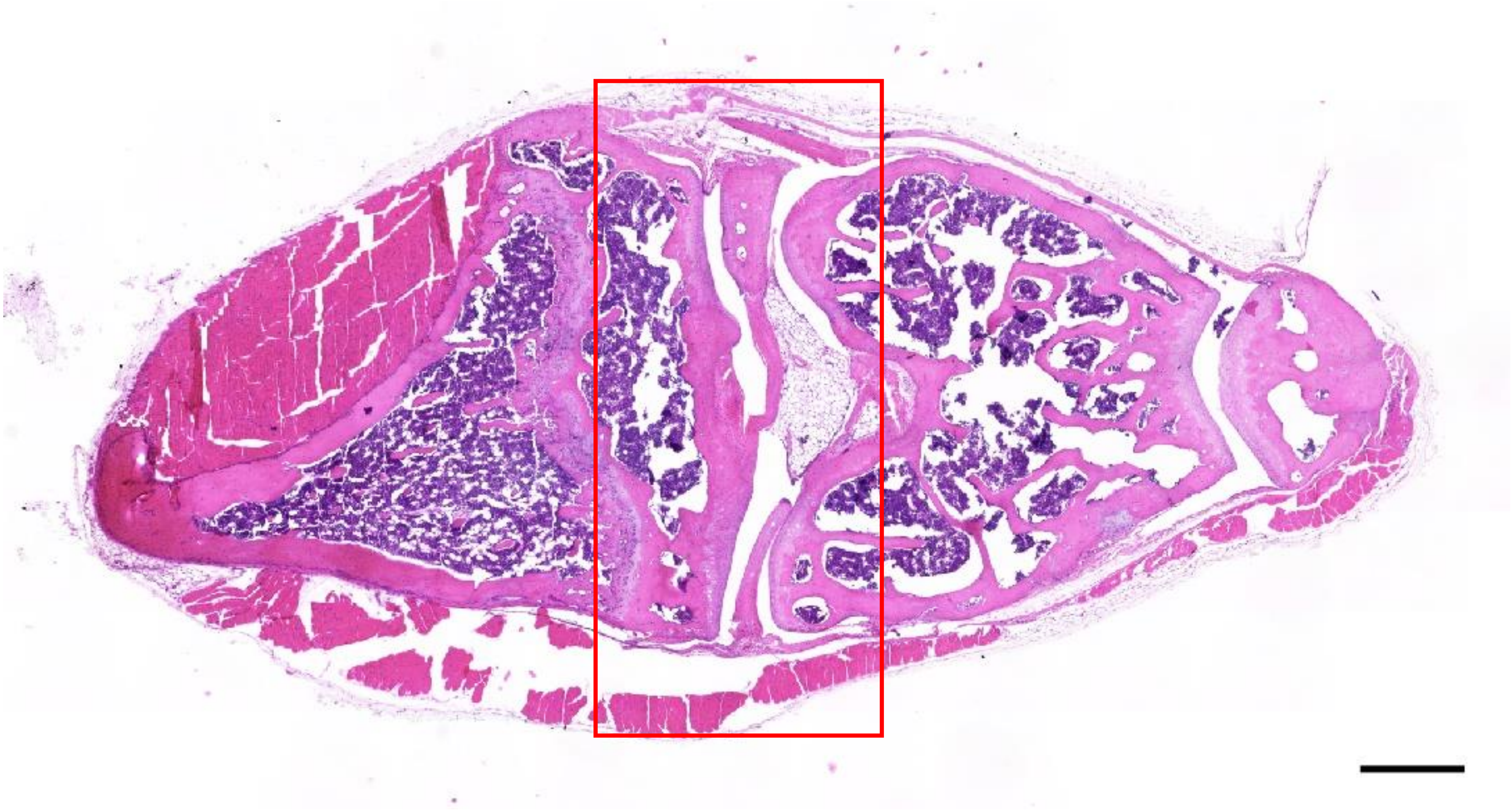

Figure 4G ***Pggt1b***$^{\Delta/\Delta}$ ***MefV*** $^{+/+}$

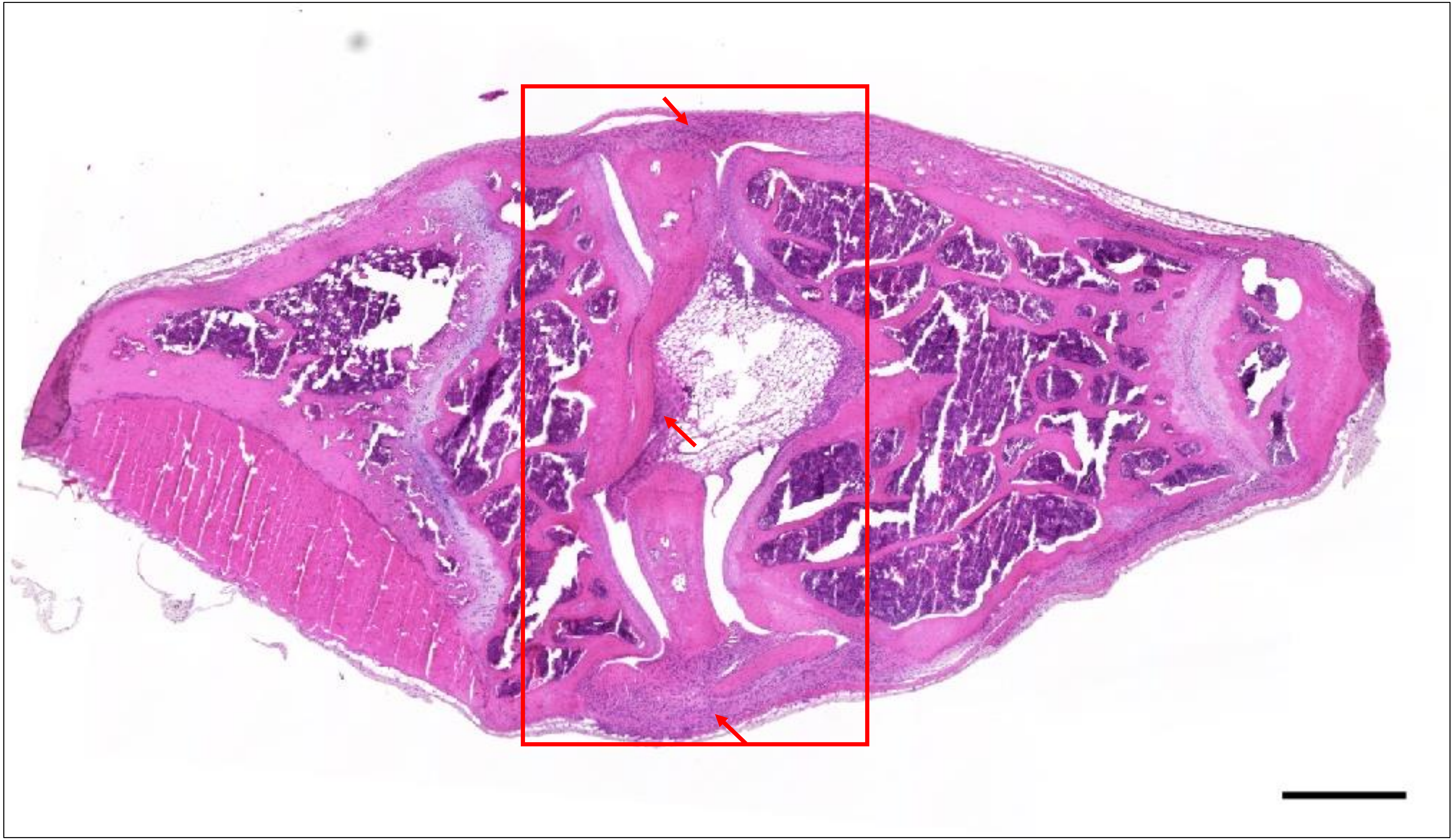

Figure 4G ***Pggt1b*** $^{\Delta/\Delta}$ ***MefV*** $^{-/-}$

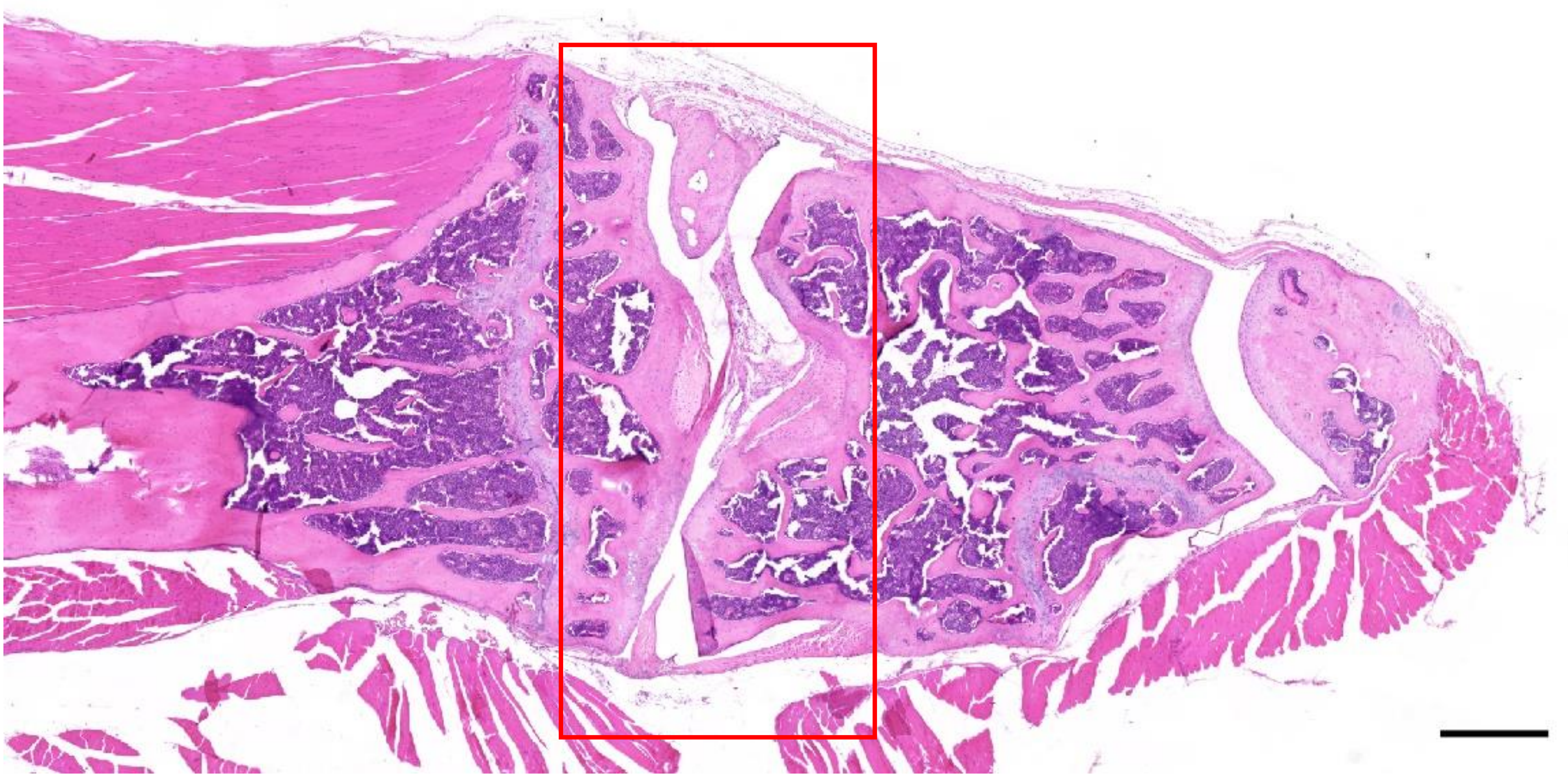

Supplement: Supplementary file 5 — Source data Fig. 4 [file 44321_2025_298_MOESM5_ESM.zip › Source Data_Figure 4/4G/SD4G.pdf]
