# Supplementary material for: Pyrin inflammasome-driven erosive arthritis caused by unprenylated RHO GTPase signaling
Source: EMBO Mol Med. 2025 Aug 29;17(10):2691–712. doi: 10.1038/s44321-025-00298-0 (PMC12514176; doi:10.1038/s44321-025-00298-0)

Figure EV1A

Pggt1b$^{+/+}$ *Tnfr1* $^{+/+}$

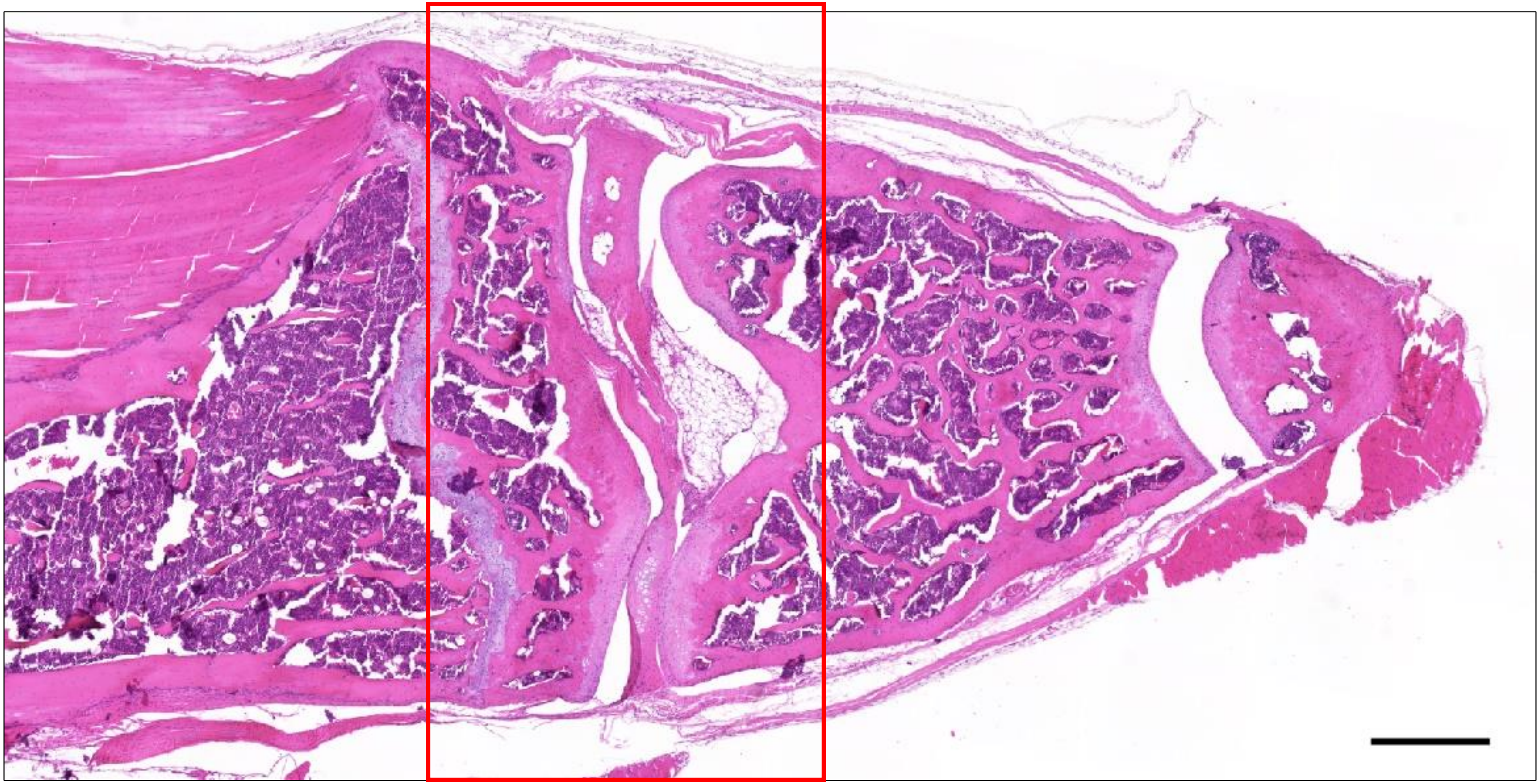

Figure EV1A *Pggt1b*$^{\Delta/\Delta}$ *Tnfr1* $^{+/+}$

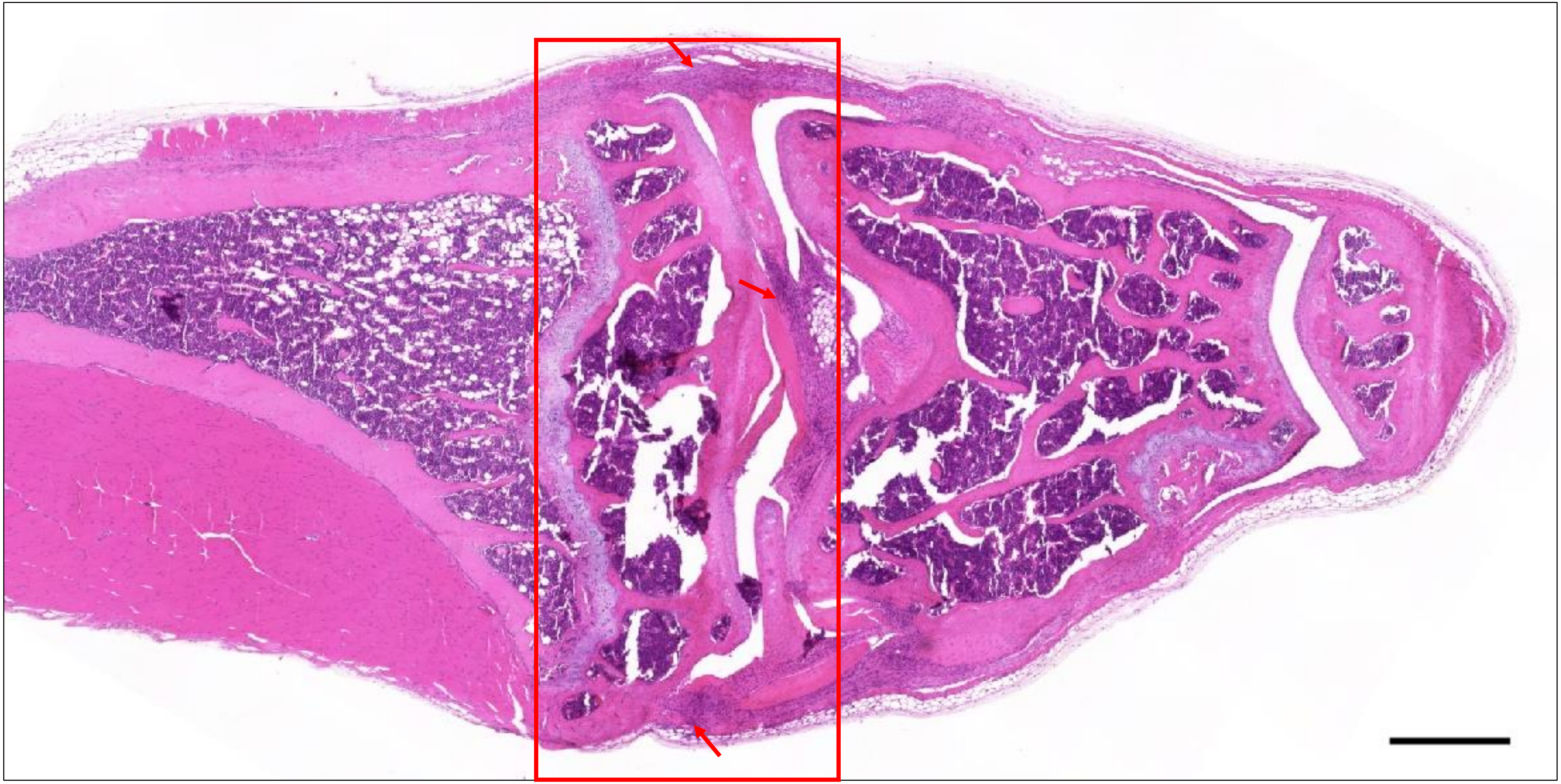

Figure EV1A ***Pggt1b$^{\Delta/\Delta}$ Tnfr1$^{-/-}$***

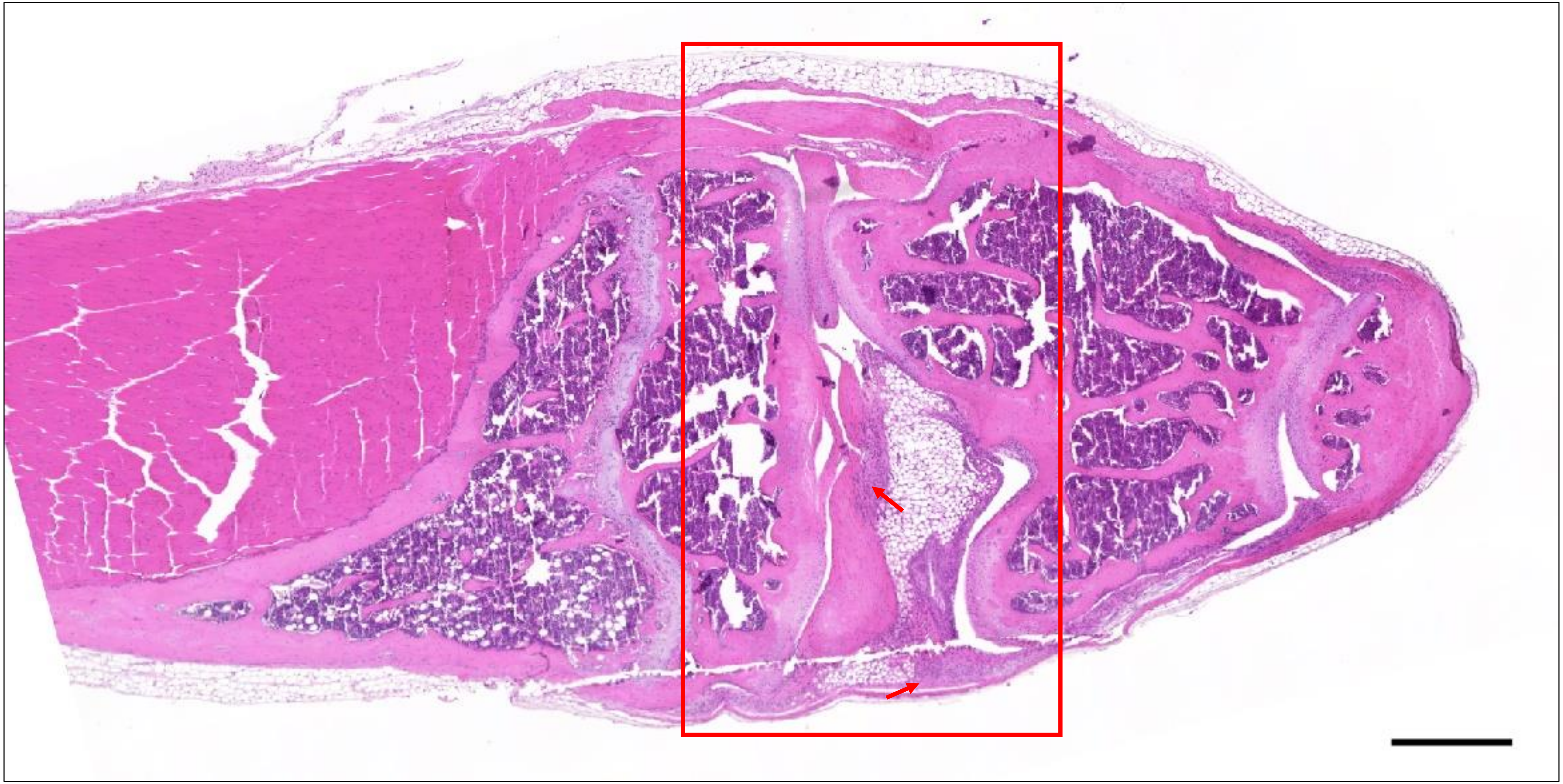

Supplement: Supplementary file 9 — Figure EV1 Source Data [file 44321_2025_298_MOESM9_ESM.zip › Source Data_Figure EV1/EV1A/SDEV1A.pdf]
