# Supplementary material for: Pyrin inflammasome-driven erosive arthritis caused by unprenylated RHO GTPase signaling
Source: EMBO Mol Med. 2025 Aug 29;17(10):2691–712. doi: 10.1038/s44321-025-00298-0 (PMC12514176; doi:10.1038/s44321-025-00298-0)

Figure EV4A *Pggt1b$^{+/+}$ Ripk1$^{+/+}$*

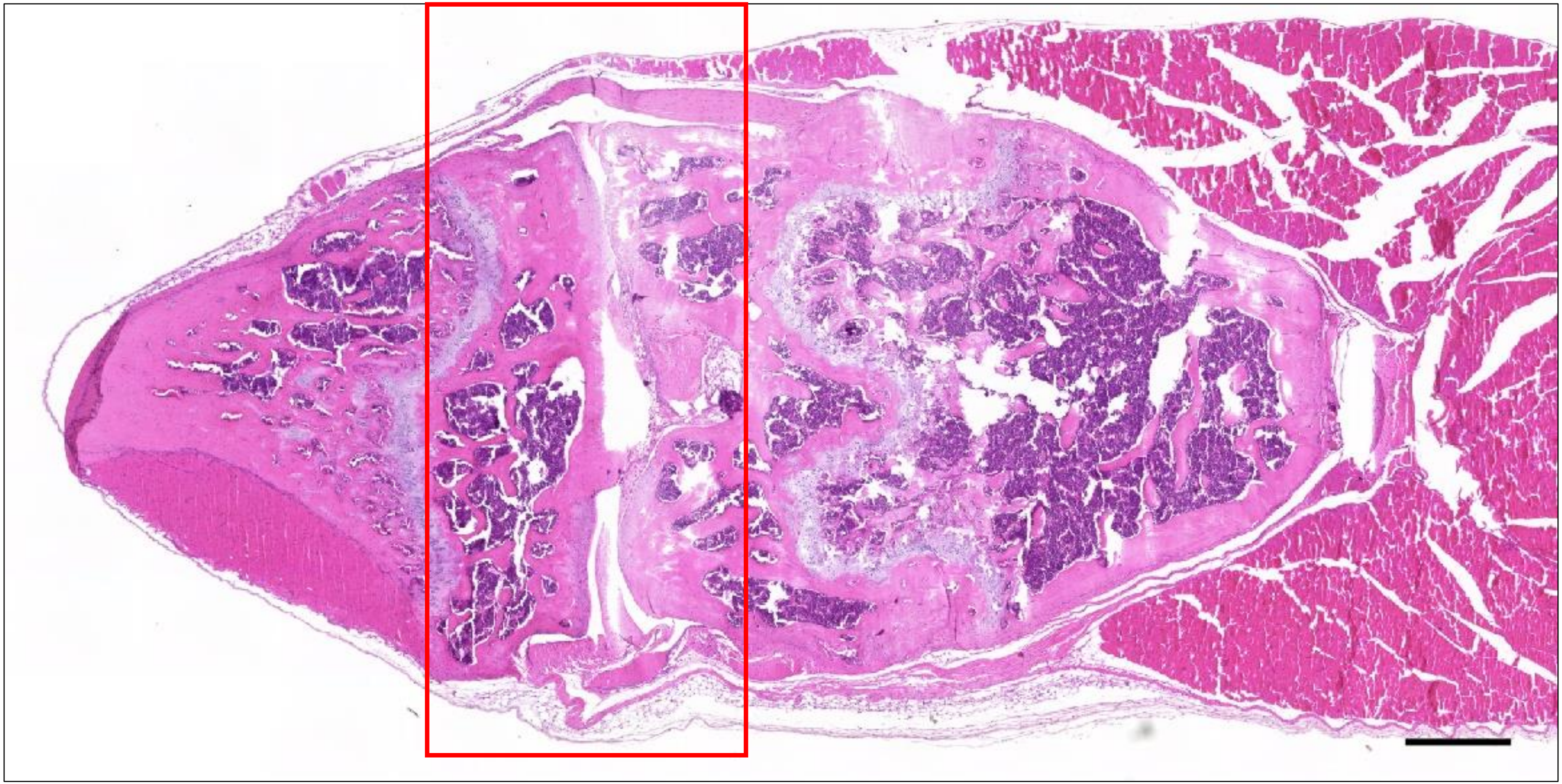

Figure EV4A *Pggt1b*$^{\Delta/\Delta}$ *Ripk1*$^{+/+}$

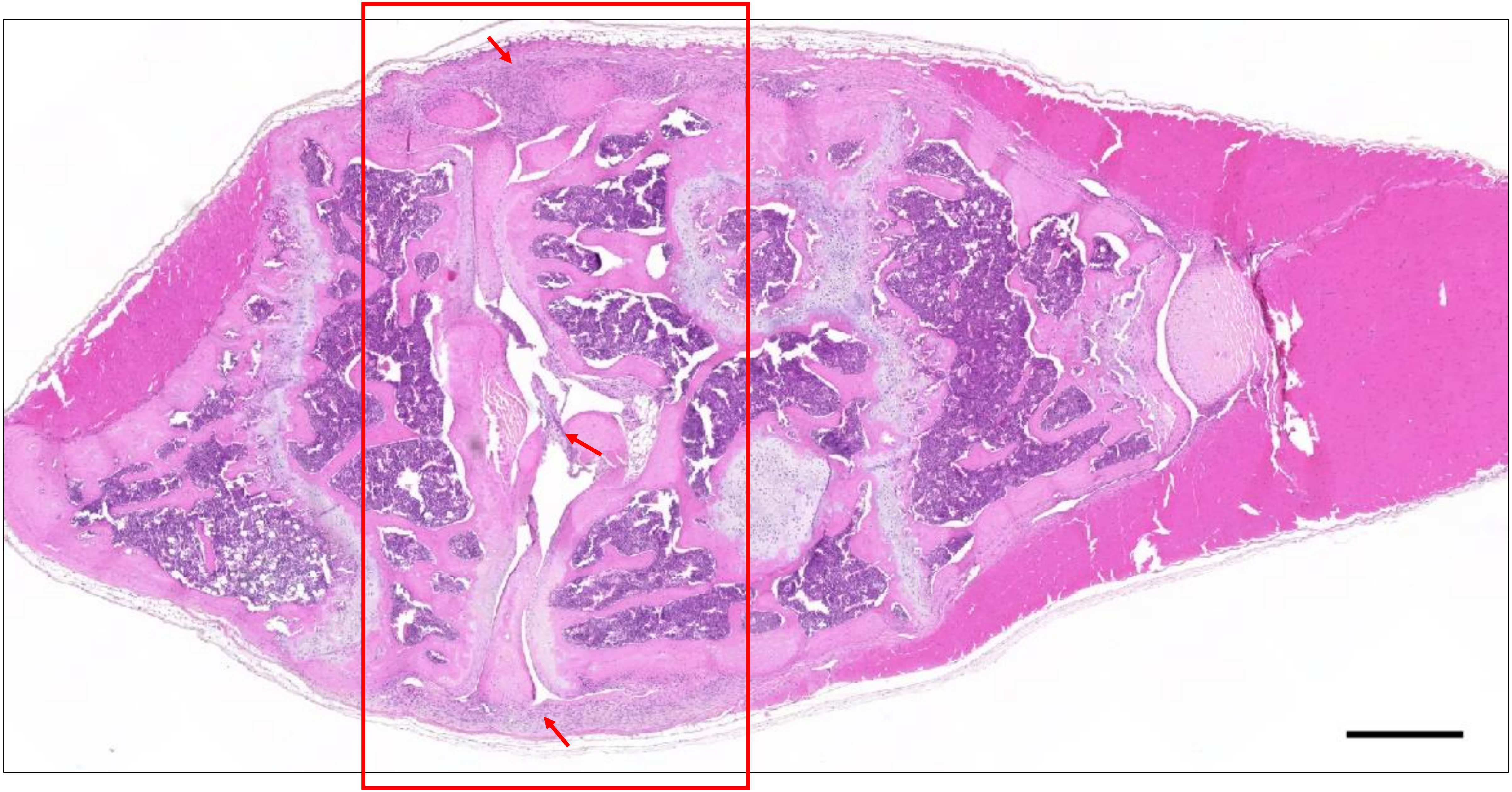

Figure EV4A *Pggt1b*$^{\Delta/\Delta}$ *Ripk1*$^{D138N/D138N}$

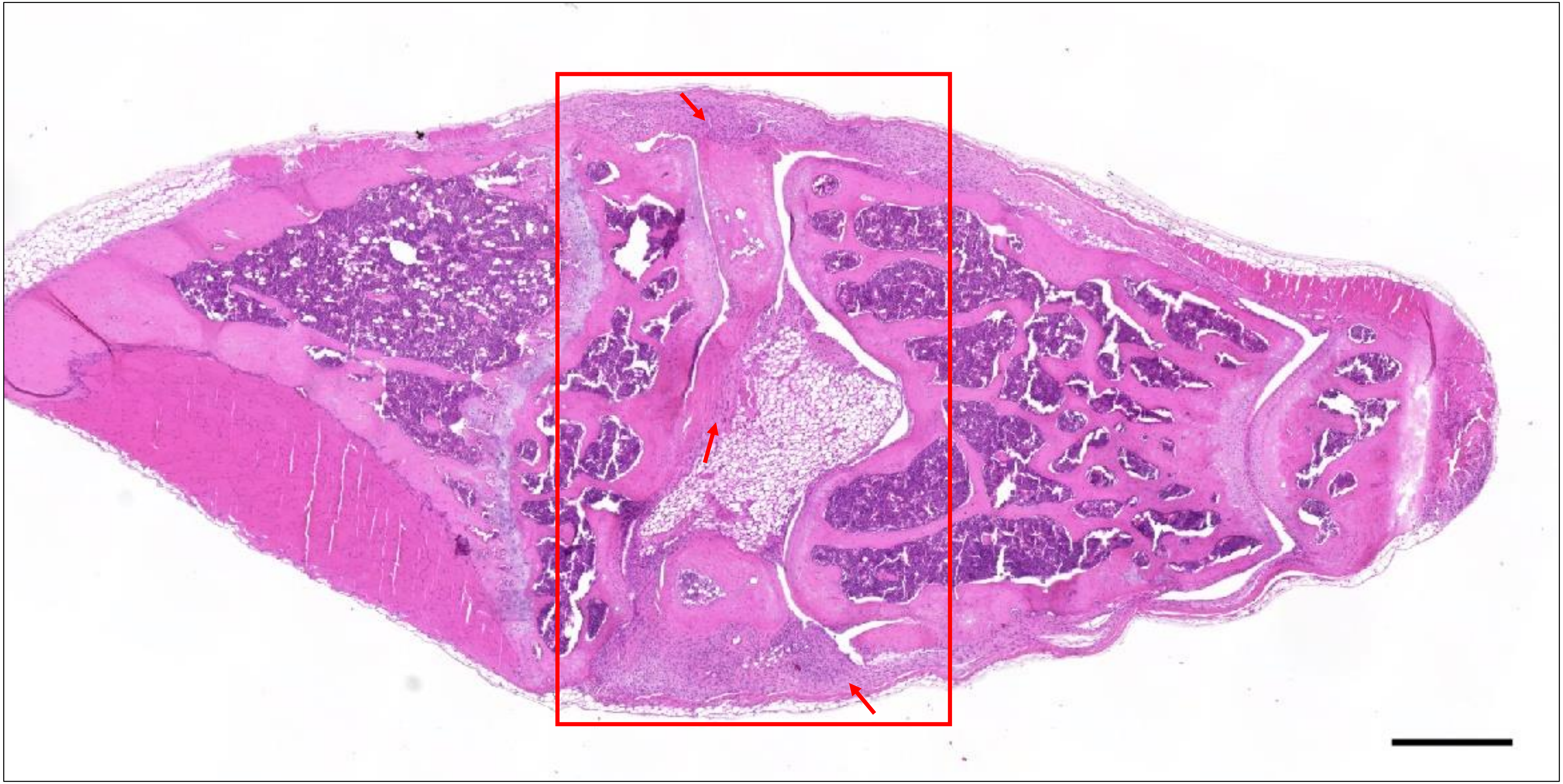

Supplement: Supplementary file 12 — Figure EV4 Source Data [file 44321_2025_298_MOESM12_ESM.zip › Source Data_Figure EV4/EV4A/SDEV4A.pdf]
