# Supplementary material for: Pyrin inflammasome-driven erosive arthritis caused by unprenylated RHO GTPase signaling
Source: EMBO Mol Med. 2025 Aug 29;17(10):2691–712. doi: 10.1038/s44321-025-00298-0 (PMC12514176; doi:10.1038/s44321-025-00298-0)

Figure EV4D

Pggt1b$^{+/+}$ *Nlrp3* $^{+/+}$

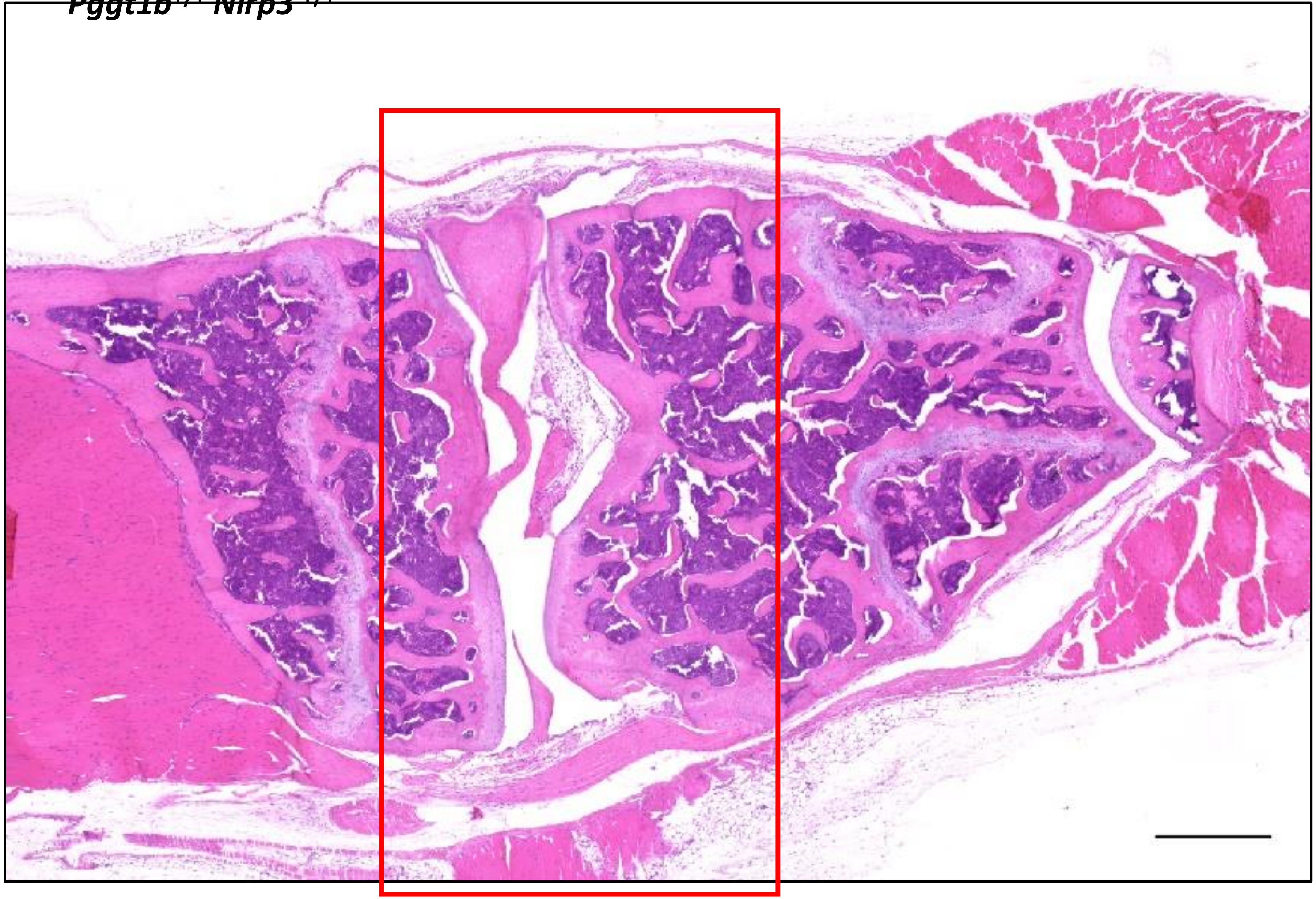

Figure EV4D *Pggt1b*$^{\Delta/\Delta}$ *Nlrp3* $^{+/+}$

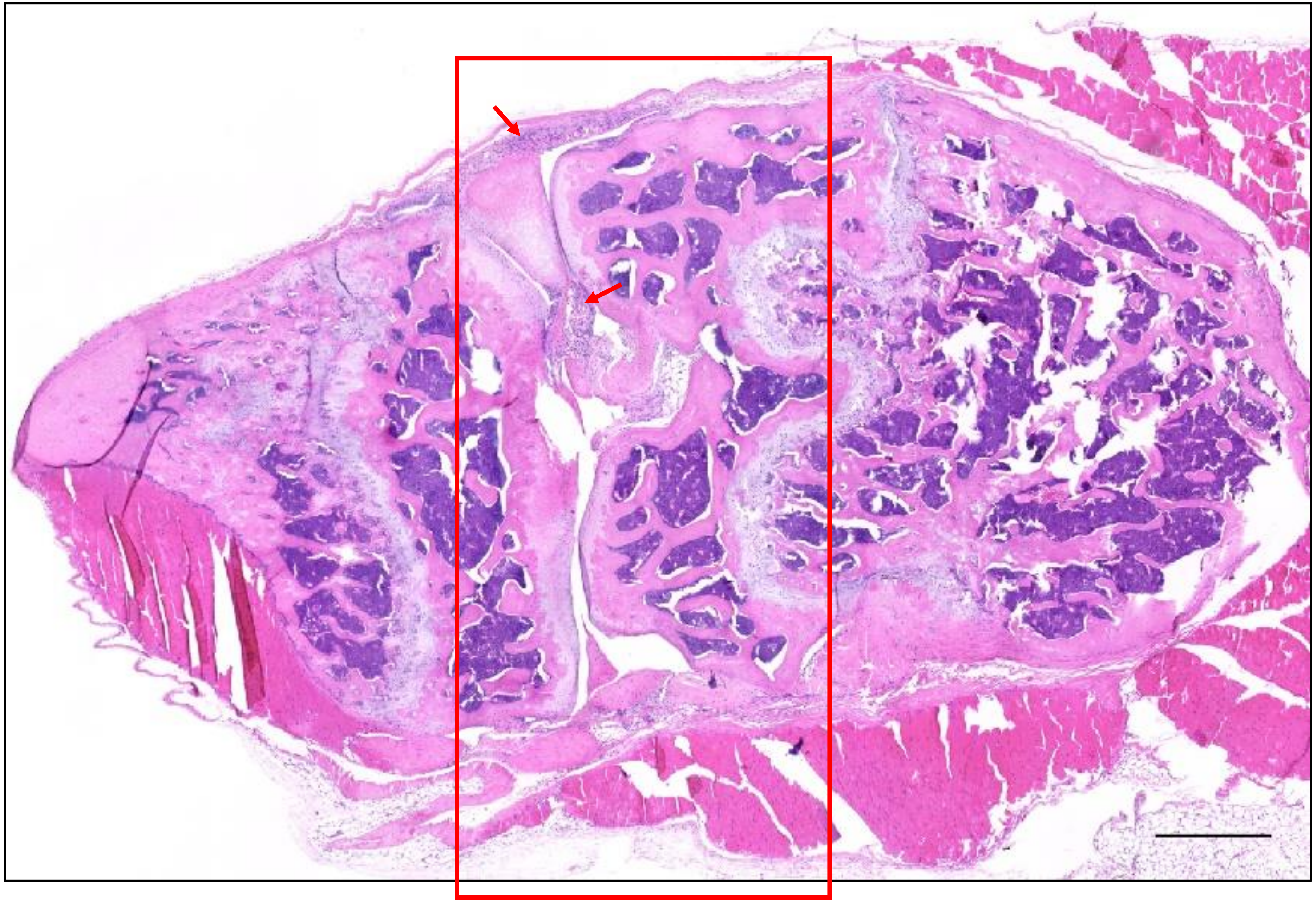

Figure EV4D ***Pggt1b***$^{\Delta/\Delta}$ ***Nlrp3*** $^{-/-}$

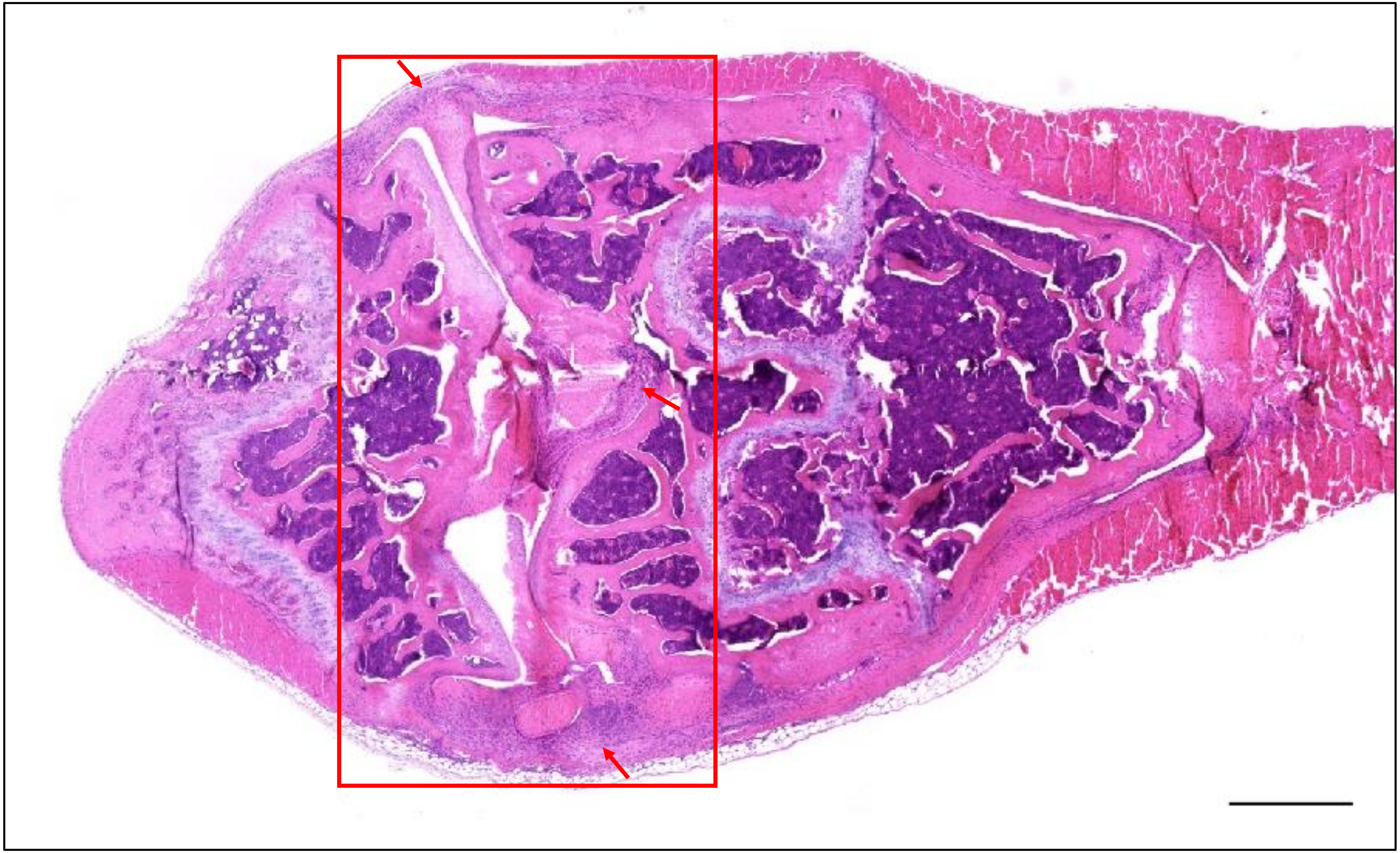

Supplement: Supplementary file 12 — Figure EV4 Source Data [file 44321_2025_298_MOESM12_ESM.zip › Source Data_Figure EV4/EV4D/SDEV4D.pdf]
