# Supplementary material for: Pyrin inflammasome-driven erosive arthritis caused by unprenylated RHO GTPase signaling
Source: EMBO Mol Med. 2025 Aug 29;17(10):2691–712. doi: 10.1038/s44321-025-00298-0 (PMC12514176; doi:10.1038/s44321-025-00298-0)
Supplement: Supplementary file 15 — Expanded View Figures [file 44321_2025_298_MOESM15_ESM.pdf]

Expanded View Figures

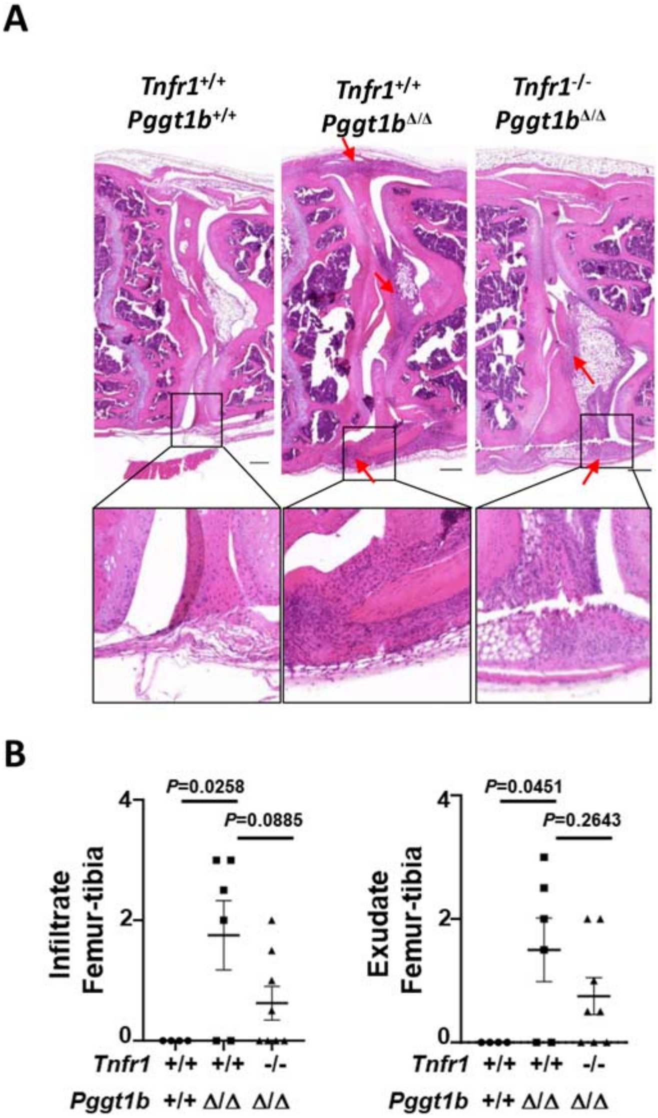

Figure EV1. TNF does not drive arthritis development in *Pggt1b*$^{\Delta/\Delta}$ mice.

(**A**) Histological images of haematoxylin and eosin-stained knee joints of 20-week-old *Pggt1b*$^{+/+}$, *Pggt1b*$^{\Delta/\Delta}$, and *Tnfr*$^{-/-}$*Pggt1b*$^{\Delta/\Delta}$ mice. Representative pictures are shown. Scalebar, 200 µm. Arrows depict inflammation, as shown by the accumulation of infiltrating leukocytes. (**B**) Histological scores for inflammation and exudate at the femur and tibia, each ranging from 0 (normal) to 3 (severely inflamed), of *Pggt1b*$^{+/+}$ ($n = 4$), *Pggt1b*$^{\Delta/\Delta}$ ($n = 6$), and *Tnfr*$^{-/-}$*Pggt1b*$^{\Delta/\Delta}$ ($n = 8$). Dots in the graphs indicate individual mice, and data are expressed as mean ± s.e.m. Significance between groups was calculated by one-way ANOVA and Dunnett's multiple comparison test. Source data are available online for this figure.

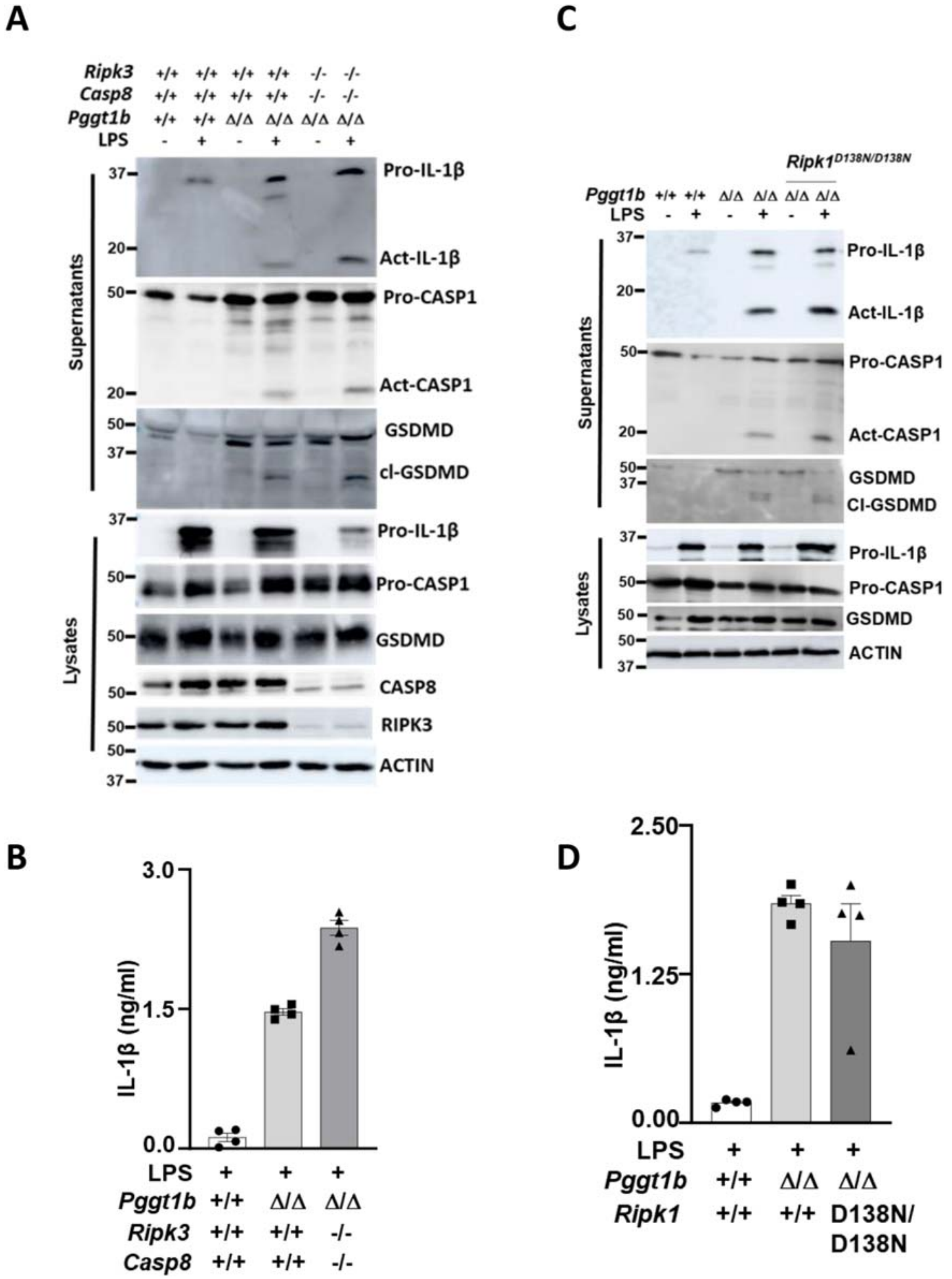
A
Ripk3 +/+ +/+ +/+ +/+ -/- -/-
Casp8 +/+ +/+ +/+ +/+ -/- -/-
Pggt1b +/+ +/+ Δ/Δ Δ/Δ Δ/Δ Δ/Δ
LPS - + - + - +
Supernatants
Lysates
Pro-IL-1β
Act-IL-1β
Pro-CASP1
Act-CASP1
GSDMD
cl-GSDMD
CASP8
RIPK3
ACTIN
C
Ripk1D138N/D138N
Pggt1b +/+ +/+ Δ/Δ Δ/Δ Δ/Δ Δ/Δ
LPS - + - + - +
Supernatants
Lysates
Pro-IL-1β
Act-IL-1β
Pro-CASP1
Act-CASP1
GSDMD
Cl-GSDMD
ACTIN
B
IL-1β (ng/ml)
3.0
1.5
0.0
LPS + + +
Pggt1b +/+ Δ/Δ Δ/Δ
Ripk3 +/+ +/+ -/-
Casp8 +/+ +/+ -/-
D
IL-1β (ng/ml)
2.50
1.25
0.00
LPS + + +
Pggt1b +/+ Δ/Δ Δ/Δ
Ripk1 +/+ +/+ D138N/ D138N

© The Author(s)

Figure EV2. Neither apoptosis nor necroptosis mediates inflammasome activation in *Pggt1b*$^{\Delta/\Delta}$ macrophages.

(**A**) Western blots showing levels of pro- and active-IL-1β, pro- and active-CASP1 and full length and cleaved (cl) GSDMD in supernatants and lysates from BMDMs isolated from *Pggt1b*$^{+/+}$, *Pggt1b*$^{\Delta/\Delta}$, and *Casp8*$^{-/-}$*Ripk3*$^{-/-}$*Pggt1b*$^{\Delta/\Delta}$ mice either stimulated or not with LPS for 8 h. Actin was used as a loading control. (**B**) IL-1β levels in supernatants from BMDMs, isolated from *Pggt1b*$^{+/+}$ ($n = 4$ biological replicates), *Pggt1b*$^{\Delta/\Delta}$ ($n = 4$) and *Casp8*$^{-/-}$*Ripk3*$^{-/-}$*Pggt1b*$^{\Delta/\Delta}$ ($n = 4$) mice after treatment with LPS for 8 h. (**C**) Western blots showing levels of pro- and active-IL-1β, pro- and active-CASP1 and full length and cleaved (cl) GSDMD in supernatants and lysates from BMDMs isolated from *Pggt1b*$^{+/+}$, *Pggt1b*$^{\Delta/\Delta}$ and *Ripk1*$^{D138N/D138N}$*Pggt1b*$^{\Delta/\Delta}$ mice either stimulated or not with LPS for 8 h. Actin was used as a loading control. (**D**) IL-1β levels in supernatants from BMDMs, isolated from *Pggt1b*$^{+/+}$ ($n = 4$ biological replicates), *Pggt1b*$^{\Delta/\Delta}$ ($n = 4$), and *Ripk1*$^{D138N/D138N}$*Pggt1b*$^{\Delta/\Delta}$ ($n = 4$) mice after treatment with LPS for 8 h. Significance between groups was calculated by one-way ANOVA with Dunnett's multiple comparison test. Source data are available online for this figure.

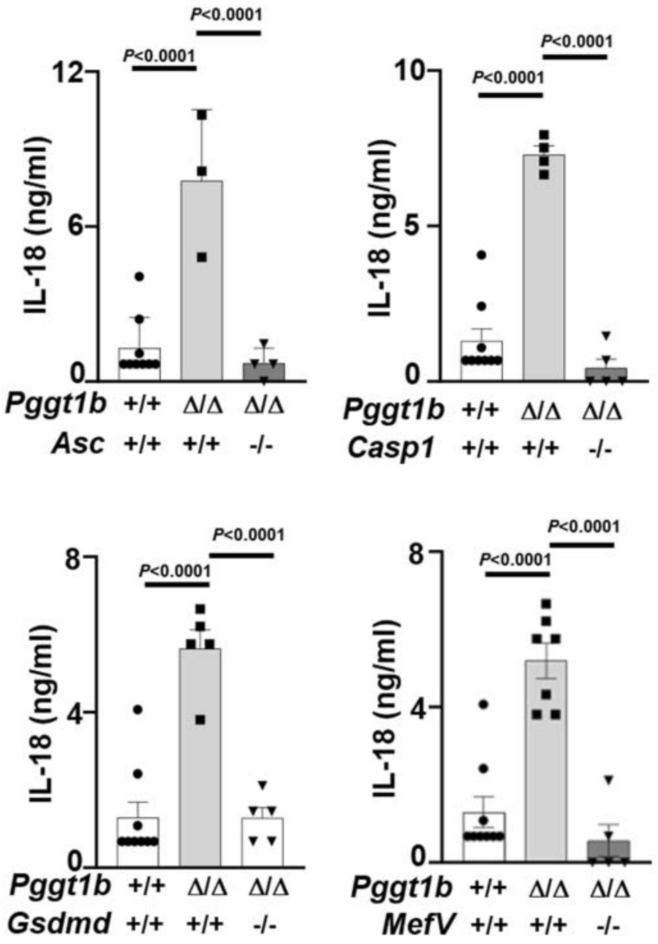

Figure EV3. Pyrin-mediated GSDMD signaling drives arthritis development in *Pggt1b*$^{\Delta/\Delta}$ mice.

IL-18 cytokine levels in serum of *Pggt1b*$^{+/+}$ ($n = 7$ biological replicates), *Asc*$^{+/+}$*Pggt1b*$^{\Delta/\Delta}$ ($n = 3$), *Asc*$^{-/-}$*Pggt1b*$^{\Delta/\Delta}$ ($n = 4$), *Casp1*$^{+/+}$*Pggt1b*$^{\Delta/\Delta}$ ($n = 4$), *Casp1*$^{-/-}$*Pggt1b*$^{\Delta/\Delta}$ ($n = 5$), *Gsdmd*$^{+/+}$*Pggt1b*$^{\Delta/\Delta}$ ($n = 5$), *Gsdmd*$^{-/-}$*Pggt1b*$^{\Delta/\Delta}$ ($n = 5$), *Mefv*$^{+/+}$*Pggt1b*$^{\Delta/\Delta}$ ($n = 7$), and *Mefv*$^{-/-}$*Pggt1b*$^{\Delta/\Delta}$ ($n = 5$). Data are expressed as mean ± s.e.m. Significance between groups was calculated by one-way ANOVA and Dunnett's multiple comparison test. Source data are available online for this figure.

© The Author(s)

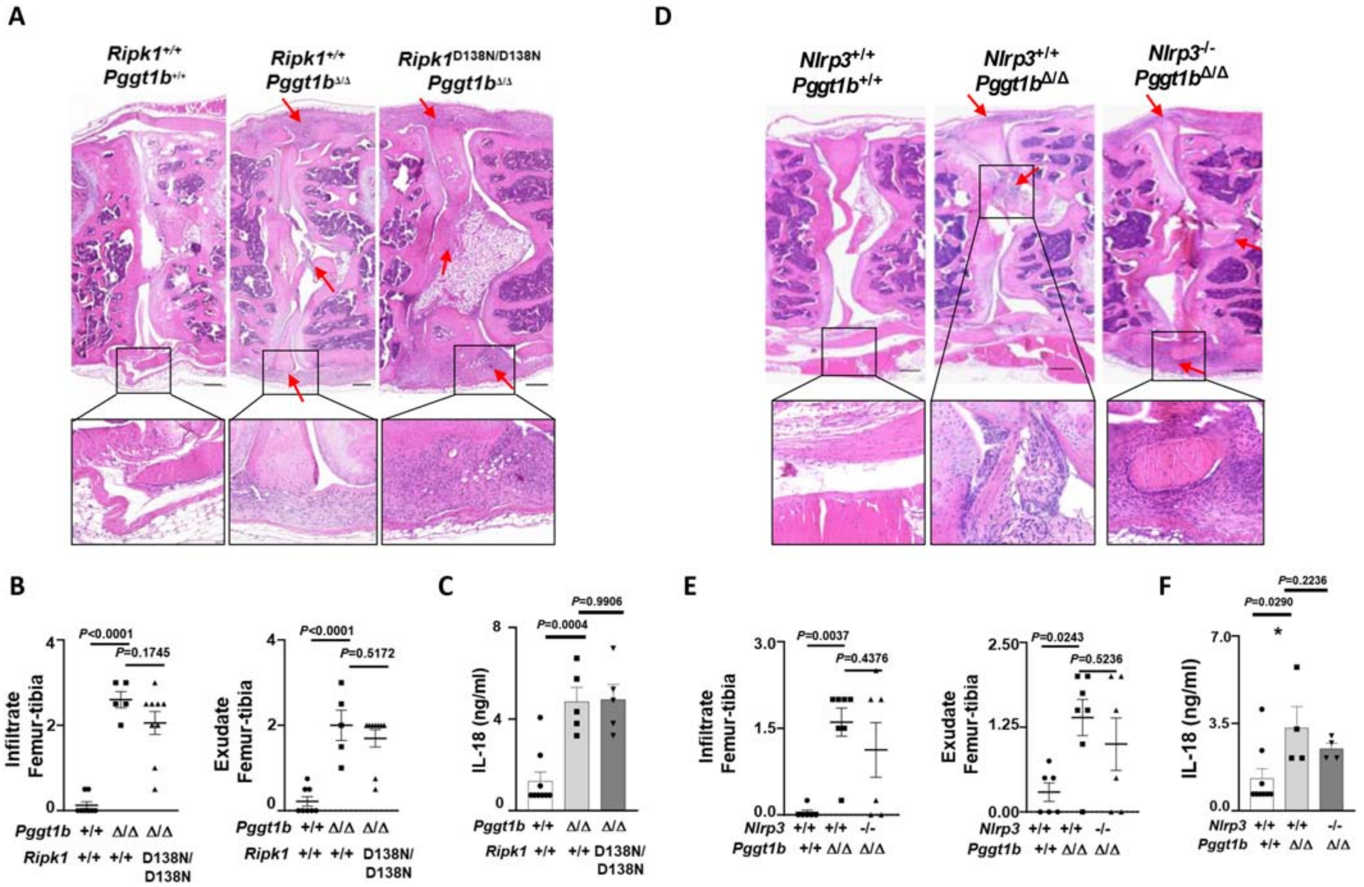

Figure EV4. RIPK1 kinase and Nlrp3 do not drive arthritis development in *Pggt1b*$^{\Delta/\Delta}$ mice.

(**A**) Histological images of haematoxylin and eosin-stained knee joints of *Pggt1b*$^{+/+}$, *Pggt1b*$^{\Delta/\Delta}$, and *Ripk1*$^{D138N/D138N}$*Pggt1b*$^{\Delta/\Delta}$ mice. Representative pictures are shown. Scalebar, 200 µm. Arrows depict inflammation. (**B**) Histological scores for inflammation and exudate at the femur and tibia, each ranging from 0 (normal) to 3 (severely inflamed), of *Pggt1b*$^{+/+}$ ($n = 9$), *Pggt1b*$^{\Delta/\Delta}$ ($n = 5$), and *Ripk1*$^{D138N/D138N}$*Pggt1b*$^{\Delta/\Delta}$ ($n = 9$) mice. (**C**) IL-18 cytokine levels in serum of *Pggt1b*$^{+/+}$ ($n = 9$ biological replicates), *Ripk1*$^{+/+}$*Pggt1b*$^{\Delta/\Delta}$ ($n = 5$) and *Ripk1*$^{D138N/D138N}$*Pggt1b*$^{\Delta/\Delta}$ ($n = 5$) mice. (**D**) Histological images of haematoxylin and eosin-stained knee joints of *Pggt1b*$^{+/+}$, *Pggt1b*$^{\Delta/\Delta}$, and *Nlrp3*$^{-/-}$*Pggt1b*$^{\Delta/\Delta}$ mice. Representative pictures are shown. Scalebar, 200 µm. Arrows depict inflammation. (**E**) Histological scores for inflammation and exudate at the femur and tibia, each ranging from 0 (normal) to 3 (severely inflamed), of *Pggt1b*$^{+/+}$ ($n = 6$), *Pggt1b*$^{\Delta/\Delta}$ ($n = 7$), and *Nlrp3*$^{-/-}$*Pggt1b*$^{\Delta/\Delta}$ ($n = 6$) mice. (**F**) IL-18 cytokine levels in serum of *Pggt1b*$^{+/+}$ ($n = 7$ biological replicates), *Nlrp3*$^{+/+}$*Pggt1b*$^{\Delta/\Delta}$ ($n = 4$) and *Nlrp3*$^{-/-}$*Pggt1b*$^{\Delta/\Delta}$ ($n = 4$). Data are expressed as mean ± s.e.m. Significance between groups was calculated by one-way ANOVA and Dunnett's multiple comparison test. Source data are available online for this figure.

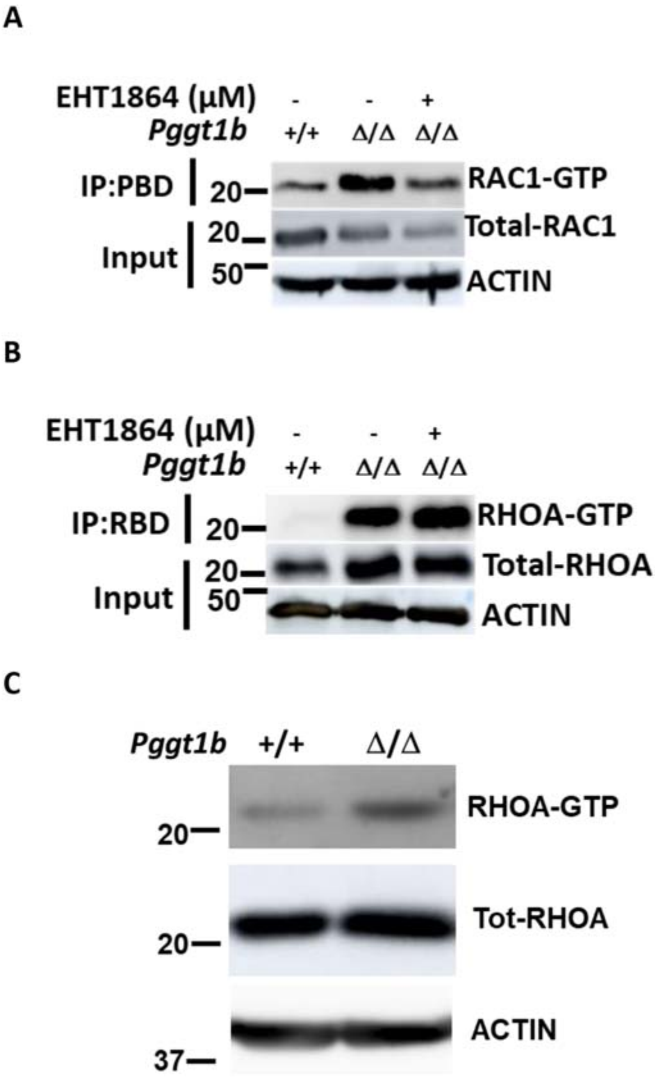

Figure EV5. RAC1 but not RHOA activates the Pyrin inflammasome in *Pggt1b*$^{Δ/Δ}$ macrophages.

(**A**, **B**) Immunoblots showing levels of total RAC1 and RAC1-GTP (**A**), and total RHOA and RHOA-GTP (**B**) in LPS-stimulated BMDMs isolated from *Pggt1b*$^{+/+}$ and *Pggt1b*$^{Δ/Δ}$ mice either treated or not with EHT1864 for 8 h. (**C**) Immunoblots showing levels of total RHOA and RHOA-GTP in lysates of *Pggt1b*$^{+/+}$ and *Pggt1b*$^{Δ/Δ}$ BMDMs after treatment with LPS for 3 h. Actin was used as a loading control. Source data are available online for this figure.

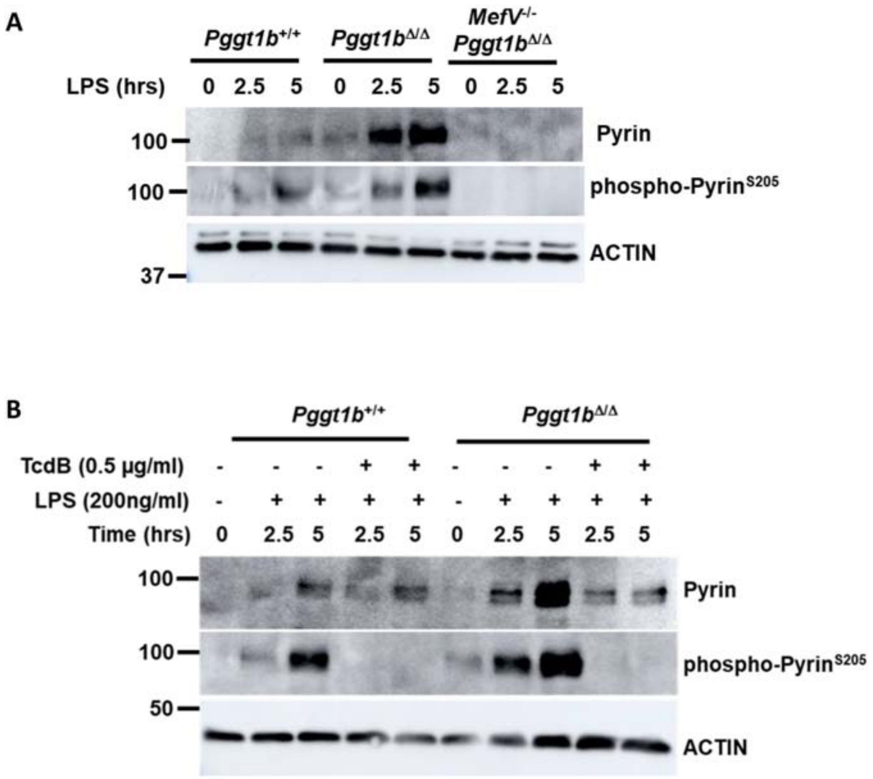

Figure EV6. *Pggt1b* deficiency does not regulate Pyrin phosphorylation levels directly.

(**A**) Immunoblots showing Pyrin and phospho (S205)-Pyrin levels in lysates of LPS-treated *Pggt1b*$^{+/+}$, *Pggt1b*$^{\Delta/\Delta}$ and *Mefv*$^{-/-}$*Pggt1b*$^{\Delta/\Delta}$ BMDMs. (**B**) Immunoblots showing Pyrin and phospho (S205)-Pyrin levels in lysates of LPS-treated *Pggt1b*$^{+/+}$ and *Pggt1b*$^{\Delta/\Delta}$ BMDMs in the presence or absence of TcdB (0.5 µg/ml). Actin was used as a loading control. Source data are available online for this figure.

© The Author(s)

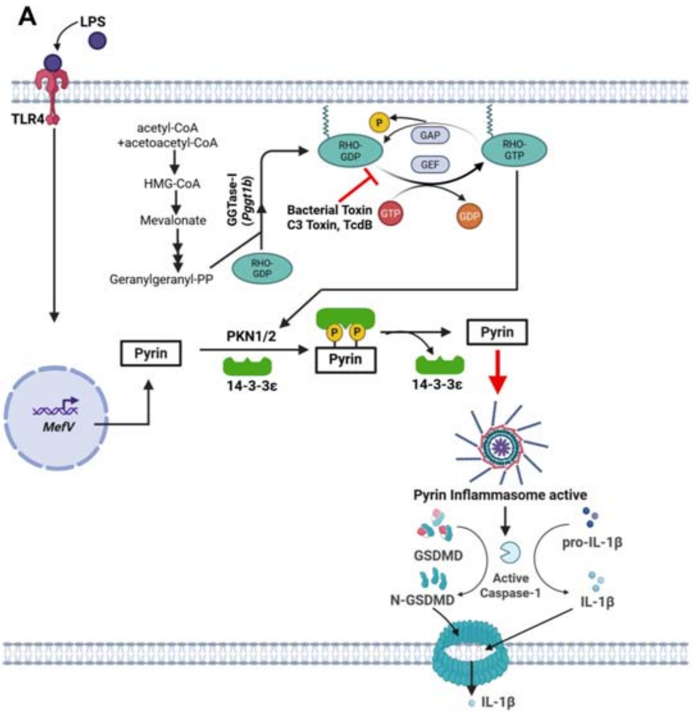

Figure EV7. Summarizing model of Pyrin inflammasome activation in toxin-treated and *Pggt1b*-deficient macrophages.

(**A**) In untreated wildtype macrophages, geranylgeranyl pyrophosphate (GGPP)—a mevalonate pathway intermediate—facilitates the prenylation of RHO family GTPases, enabling their membrane localization and activation. Active RHO-GTP proteins engage effectors and activate kinases PKN1/2, which phosphorylate Pyrin and promote its binding to 14-3-3 proteins, thereby suppressing inflammasome activation. Bacterial toxins that inactivate RHO proteins prevent PKN1/2-mediated Pyrin phosphorylation, resulting in inflammasome assembly and IL-1β production. (**B**) In *Pggt1b*-deficient macrophages, impaired prenylation disrupts RHO protein membrane targeting and signaling. This leads to Pyrin inflammasome assembly with ASC and CASP1, cleavage of GSDMD and proIL-1β, and secretion of active IL-1β. Secreted IL-1β signals via the IL-1 receptor to upregulate *Mefv* transcription, thereby increasing Pyrin inflammasome activation. GTP guanine triphosphate, GDP guanine diphosphate, GEF guanine nucleotide exchange factors, GAP GTPases-activating proteins, TLR4 Toll-like receptor 4.

 © The Author(s)
